# The indane diastereoisomers, PH2 and PH5: divergence between their effects in delayed-type hypersensitivity models and a model of colitis

**DOI:** 10.1111/jphp.12846

**Published:** 2017-10-23

**Authors:** Neil H Frankish, Brendan McHale, Helen Sheridan

**Affiliations:** School of Pharmacy and Pharmaceutical Technology, Trinity College Dublin, Dublin, Ireland; MSD, Red Oak North, South County Business Park, Leopardstown, Dublin, Ireland

**Keywords:** bi-indanes, colitis, delayed-type hypersensitivity, inflammatory bowel disease

## Abstract

**Objectives:**

Compounds PH2 and PH5 are distereoisomers of novel indane compounds, synthesised as analogues of secondary metabolites of the fern, *Onychium*. In this study, we compare their effects on a variety of inflammatory models.

**Methods:**

In an effort to extend our knowledge of their anti-inflammatory profile, we have investigated their activity in two models of delayed-type hypersensitivity (DTH); the methylated bovine serum albumin model (mBSA) and the oxazolone contact hypersensitivity (CHS) model, on IL2 release from Jurkat cells and in the dextran sulphate sodium (DSS) murine model of inflammatory bowel disease.

**Key findings:**

Both diastereoisomers are equipotent in reducing paw swelling in the mBSA model and in inhibiting interleukin (IL) 2 release from Jurkat cells. They are equally ineffective in the oxazolone contact hypersensitivity model (CHS). Only the diastereoisomer, PH5, protects against DSS-induced colitis and of its two enantiomers, only the S,S-enantiomer, PH22, possesses this activity. PH2 is ineffective in the DSS model.

**Conclusions:**

The results suggest that the beneficial effect of PH5, and its enantiomer PH22, in the DSS model is a consequence of an action on a target specific to the colitis model. The implications of such data suggest an unknown target in this disease model that may be exploited to therapeutic advantage.

## Introduction

Ferns belonging to the *Onychium* family (*Pteridaceae*) have a substantial history of use in traditional Asian medicine to treat gastrointestinal conditions.^[1,2]^ Initially, our previous studies were focused on *Onychium* metabolites with the indane skeleton, which feature in a range of molecules in clinical use and which display significant and diverse biological activity.^[3–5]^ We have established that monomeric indanes, related to the fern metabolites^[6–8]^ together with synthetic 1,2-coupled dimers^[9,10]^ demonstrate smooth muscle relaxant activity and also inhibited histamine release stimulated by compound 48/80 from rat peritoneal mast cells. Initially, we considered this class of compounds, which demonstrated mast cell stabilisation coupled with bronchodilatory activity, as potential treatments for asthma.^[9]^ However, it became evident that this approach was not viable, as there seemed to be an inverse relationship between smooth muscle relaxant activity and mast cell stabilisation.^[11]^ However, the finding that compounds PH2 and PH5 had significant anti-inflammatory activity^[12]^ suggested that other avenues of research should be pursued.

The major difficulty surrounding the development of novel therapeutic agents from traditional medicine, is that while clinical responses can be noted, much evidence is anecdotal. When an active natural product has been identified from the traditional medicine, one cannot be certain that other components have other activities or that one or more can act in concert, perhaps synergistically. Where biological activity is confirmed in the laboratory, the question of an agent's mode of action is left open. This makes the directed synthesis and screening of synthetic analogues of natural products difficult. The lack of a specific enzyme inhibition or ligand-binding assay mitigates against high-throughput screening and needs must one revert to phenotypic response assays which are slow and relatively more expensive. When a relevant tissue or cell type cannot be identified, then only *in vivo* models can serve. Consequently, we have characterised for the first time the biological activity of these novel di-indanes on a model of cytokine release and in a range of *in vivo* inflammatory models.

## Materials and Methods

### Jurkat cells

Jurkat T cells were cultured in RMPI-1640 medium containing 10% foetal bovine serum, penicillin/streptomycin and l-glutamine in T-75 cm^2^ flasks at 37°C and 5% CO_2_. Cells were seeded into new culture flasks at a density of 1–3 × 10^5^ cells/ml and maintained at a density of 0.5–1 × 10^6^ cells/ml.

#### Anti-TCR/CD28-mediated activation of Jurkat cells

Experiments involving anti-TCR/CD28-mediated activation of Jurkat T lymphocytes were carried out on 24-well cell culture plates. The plates were coated with a 1/100 dilution of rabbit anti-mouse IgG in sterile PBS (1×) and incubated overnight at 4°C. Unbound antibody was aspirated and the wells were gently rinsed with warm sterile PBS. The PBS was aspirated and the wells were coated with a 1/100 dilution of anti-CD3 antibody (OKT-3) and a 1/200 dilution of anti-CD28 antibody (both antibodies premixed prior to addition to the plates) in sterile PBS. The plates were then incubated for 1–2 h at 37°C. Unbound antibody was aspirated and the wells were gently rinsed with warm PBS. The PBS was left on the plate until the Jurkat cells were ready to be added to the plate. The Jurkat cells were harvested from the T-75 cm^2^ cell culture flasks and resuspended at a density of 0.5 × 10^6^ cells/ml. The cells were then preincubated with the compounds or with an equivalent volume of drug vehicle control (DMSO) for 30 min prior to adding the cell suspension to the antibody-coated plates. Ciclosporin A (1 μm) was used as a positive control to inhibit IL-2 production from activated T cells and was preincubated with the cells as described above in parallel. The cells were then incubated on the plates with the acti-TCR/CD28 antibodies for 24 h at 37°C and 5% CO_2_ in a humidified atmosphere. After 24 h, the cell suspension was removed from the plates and the cells were pelleted by centrifugation. The cell culture supernatant was saved and analysed for secreted IL-2 by ELISA. The cell pellet was saved and cell viability was measured using Acridine Orange/Ethidium Bromide cell viability staining solution.

#### PHA/PMA-mediated activation of Jurkat cells

Jurkat cells were resuspended at a concentration of 0.5 × 10^6^ cells/mL, and the compounds at the desired concentration or an equivalent volume of drug vehicle control (DMSO) were added to the cells and incubated for 30 min. Ciclosporin A (1 μm) was used as a positive control to inhibit IL-2 production from activated T cells and was preincubated with the cells as described above in parallel. PHA and PMA were added to the cell suspension (10 and 10 ng/mL respectively), and the cells were plated onto uncoated 24-well plates. The cells were incubated on the plates for 24 h at 37°C and 5% CO_2_ in a humidified atmosphere. After 24 h, the cell suspension was removed from the plates and the cells were pelleted by centrifugation. The cell culture supernatant was saved and analysed for secreted IL-2 by ELISA.

#### Measurement of cell viability

The cell pellet was resuspended in 20 μL of Acridine Orange/Ethidium Bromide staining solution. A portion of this cell suspension was placed on a haemocytometer and analysed under a fluorescence microscope. Viable cells appeared green whilst non-viable cells had a characteristic orange appearance. The ratio of viable cells: non-viable cells were calculated for each activation time point/drug treatment.

The cell culture supernatants were analysed for IL-2 by ELISA (96-well plates format) using the Human IL-2 ELISA kit from R&D Systems Europe according to the manufacturer's instructions using a 96-well plates reader (Titertek Multiscan; Medical Supply Company, Dublin, Ireland) at 450 nm.

### Inflammatory *in vivo* experiments

#### The methylated bovine serum albumin paw swelling model

The mBSA delayed-type hypersensitivity (DTH) model was a modification of that described by Tarayre and co-workers ^[13]^ . CD-1 mice (25–35 g) were anaesthetised with halothane and immunised i.d. with mBSA/Freunds complete adjuvant (FCA) containing *Mycobacterium butyricum* (FCA(B)) emulsion at four sites (62.5 μg/25 μl at each site) on the shaved chest on day 1. The mBSA/FCA(B) is prepared by emulsifying equal volumes of mBSA and FCA(B) solutions (the mBSA solution was first prepared in sterile isotonic saline at a concentration of 5 mg/mL). On days 8 and 9, mice were dosed intraperitoneally with either 1% carboxymethylcellulose (CMC), Ciclosporin (50 mg/kg in 1% CMC) or test compound (10 mg/kg in 1% CMC). Two hours after the second intraperitoneal dose (day 9), anaesthetised mice were challenged, by injecting mBSA in saline s.c. in the dorsal surface of the right hind paw and s.c. in the left hind paw with saline alone (20 μL each injection). Twenty-four hours later, mice were sacrificed by cervical dislocation, and the swelling of each paw measured in triplicate with a plethysmometer (Model 7140 Plethysmometer; Ugo Basile, Monvalle VA, Italy).

Each test group consisted of six mice. Paw volume measurements (mL) were used to calculate the increase in the mBSA-challenged paw compared with the saline-injected contralateral paw of each mouse, as follows: paw swelling (% difference) = (mBSA-injected paw volume (mL)) – (saline-injected paw volume (mL)) ×100%/Saline-injected paw volume (mL).

#### The oxazolone contact hypersensitivity model (CHS) in Balb/C mice

The oxazolone CHS model was a modification of that of Xu and co-workers.^[14]^ Female Balb/C mice (30–40 g) were sensitised with 20 μl of 2% oxazolone in acetone on each ear (10 μL on the inner and outer aspects of both ears). All mice were challenged with 20 μL of 2% oxazolone in acetone only on the right ear; again, 10 μL on both the inner and outer aspects of the ear. Immediately following this, the mice were treated with test compound. The test compound was administered in exactly the same way as the oxazolone, with 10 μL on each side of the right ear. All compounds were prepared in acetone at a concentration of 15 mg/ml; 300 μg/ear. For the positive control group, acetone was administered. Mice were killed 24 h later, and the percentage increase in ear swelling was calculated. This was performed in two ways. The thickness of the unchallenged left ear and the challenged right ear were measured using a micrometer caliper (μm). The increase in weight of both ears was also measured using a 5 mm biopsy punch (mg). The percentage increase in oedema was then calculated for both weight and thickness by expressing the difference between the unchallenged left ear and the challenged right ear as a percentage of the left ear control.

#### Dextran sulphate sodium colitis model

Specific pathogen-free female Balb/c mice, 6–8 weeks of age, were obtained from a commercial supplier (Harlan, UK). Mice were fed irradiated diet and housed in individually ventilated cages (Tecniplast, UK) under positive pressure. All compounds and experimental groups were randomly alphabetically labelled. Throughout experiments, all data recording was performed in a blind manner. The codes on boxes/groups were not broken until after the data were analysed. A 5% DSS solution was prepared in drinking (tap) water, with fresh DSS solution provided every second day. Compounds were injected on days 0–7, and mice were culled on day 8. The mice were checked each day for morbidity and the weight of individual mice recorded. Induction of colitis was determined by weight loss, faecal blood, stool consistency, and, upon autopsy, length of colon and histology. Approx. 1 cm of distal colon was removed for histology. The rest of the colon was recovered, snap-frozen and stored at −200°C for immunological analysis.

##### Disease activity index

To quantify the severity of colitis, a disease activity index (DAI) was determined based on previous studies of DSS-induced colitis^[15]^. DAI was calculated for individual mice on each day based on weight loss, occult blood and stool consistency as described elsewhere.^[16]^ A score was given for each parameter, with the sum of the scores used as the DAI, the maximum score being 12.

##### Histological grading of DSS-induced colitis

Sections of distal colon were fixed in 10% formaldehyde-saline. Tissue was paraffin embedded and 5 μm serial sections cut. Slides were stained with haematoxylin and eosin. Histological grading was performed blind and was on a scoring system modified as described,^[17]^ assessing severity of cell infiltration (0; none, 1; slight with dispersed cell infiltrate, 2; moderate with increased cell infiltrate forming occasional immune cell foci, 3; severe with large areas of immune cell infiltrate causing loss of tissue architecture), the extent of damage (0; none, 1; mucosal, 2; mucosal and submucosal, 3; transmural) and crypt damage (0; none, 1; basal 1/3 damaged, 2; basal 2/3 damaged, 3; only surface epithelium intact, 4; loss of entire crypt and epithelium). The combined score from each feature graded was calculated for individual mice with the maximum score being 10.

After removal of approx. 1 cm of colon for histology, the remainder of the colon was snap-frozen and stored. Individual colon tissue samples were thawed, gut contents removed, chopped finely and homogenised. The protein concentration of the supernatant was determined. Colon levels of myeloperoxidase (MPO) were determined essentially as described previously.^[18]^ Cytokines in colon supernatants were analysed using conventional sandwich ELISAs. Levels of cytokines and MPO are expressed relative to colon protein.

### Materials

Oxazolone, *M. butyricum*, CMC, Ciclosporin A (Cic. A), PBS and trypan blue stain, phytohaemagglutinin (PHA), phorbol myristate acetate (PMA), DMSO were obtained from Sigma Aldrich, Arklow, Ireland. DSS (35–50 000 kDa) was purchased from ICN. Coating antibodies, standards and detecting antibodies were obtained from BD, Dublin, Ireland or R&D Systems, Abingdon, UK. Human IL-2 ELISA kit sourced from R&D Systems Abingdon, UK. T-cell leukaemic line Jurkat E6.1 was obtained from ATCC, LGC Standards, Middlesex, UK. Anti-CD28 monoclonal antibody (stimulating) Ancell (UK). RPMI-1640 cell culture medium, Foetal Bovine Serum, Penicillin/Streptomycin, L-Glutamine, 10× Phosphate-Buffered Saline (PBS) from Gibco BRL, UK.

Compound PH2 is one of a pair of diastereoisomers, being a racemic mixture of the two enantiomers (*R,S*/S,*R*)-2-benzyl-2,3-dihydro-2-(1H-inden-2-yl)-1H-idnen-1-ol, with PH5 being the other diastereoisomer ((*S,R*/*S,S*)-2-benzyl-2,3-dihydro-2-(1H-inden-2-yl)-1H-idnen-1-ol). Compounds PH21and PH22 are single enantiomers separated from PH5, being (*1R, 2R*) and (*1S, 2S*) -2-benzyl-2,3-dihydro-2-(1H-inden-2-yl)-1H-idnen-1-ol), respectively. Details of the synthesis, separation, characterisation and confirmation of structure by X-ray diffraction of these compounds are described elsewhere.^[11,12,19]^

A total of 123 mice were used in this study. All animal research work reported in this article have been carried out in the Comparative Medicine Unit, Trinity College Dublin, a Health Products Regulatory Authority (HPRA) approved establishment that operates in accordance with Directive 2010/63/EU and its Irish transposition S.I No 543 of 2012. Trinity College Dublin complies with the Council for International Organizations of Medical Sciences’ (CIOMS), International Guiding Principles for Biomedical Research Involving Animals, and all laws, regulations and policies governing the care and use of laboratory animals in the jurisdiction in which the research is being conducted.

Data were presented graphically and analysed statistically using Graphpad Prism software (GraphPad Software, Inc., La Jolla, CA, USA). Statistical comparisons were made by one-way ANOVA followed by Dunnett's multiple comparisons test as a post-test. Statistical significance was taken as *P*<0.05 (*).

## Results

### Effect on delayed-type hypersensitivity models of inflammation

#### mBSA mouse paw swelling

**Figure 1:**
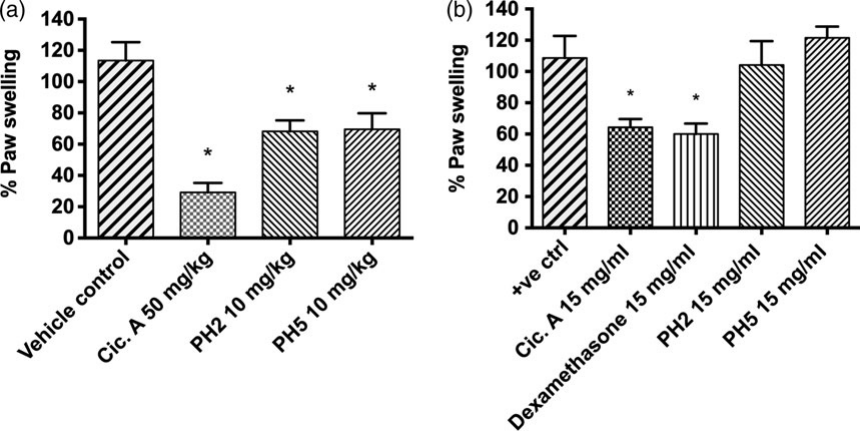
(a) Effect of PH2 and PH5 on mBSA-stimulated mouse paw swelling. (b) Effect of PH2 and PH5 on oxazolone-stimulated mouse ear swelling. Values expressed as a mean ± SEM,* n* = 7. *P<0.05.

Methylated bovine serum album-sensitised mice when challenged by the antigen after administration of saline vehicle responded by an increase in paw volume 24 h later of 113%. In contrast, those mice treated with ciclosporin A at 50 mg/kg had their paw swelling reduced to almost 30%. In comparison, both PH2 and PH5 at 10 mg/kg showed paw swelling of almost 70% of control (see Figure 1a).

#### The oxazolone contact hypersensitivity model

Challenge with 20 μL of 2% oxazolone to the ears of vehicle (acetone) only treated right ears in oxazolone-sensitised mice increased ear punch weight by 109 ± 14%. Both ciclosporin and dexamethasone (300 μg/ear) reduced ear swelling but neither PH2 nor PH5 (300 μg/ear) had any significant effect on oxazolone-induced mouse ear swelling. See Figure 1b.

### Effect on IL2 release from Jurkat cells

The Jurkat E6.1 T-cell line, which is phenotypically similar to a normal human T cell, was used to study the effect of PH2 and PH5 on IL-2 secretion. Ciclosporin A (1 μm) was used as a positive control to inhibit the production of IL-2 in all experiments. IL-2 secretion was measured following activation of Jurkat T cells with two different methods of stimulation; the first method of stimulation was a combination of antibodies to the CD3 complex in combination with the accessory signalling molecule CD28. The second method of stimulation was a combination of the mitogen PHA and phorbol ester PMA. Cell viability of Jurkat cells in the presence of PH2 and PH5 at 1 and 10 μm, either unstimulated or stimulated by anti-CD3/CD28 was between 95 and 98% (*n*=3).

**Figure 2:**
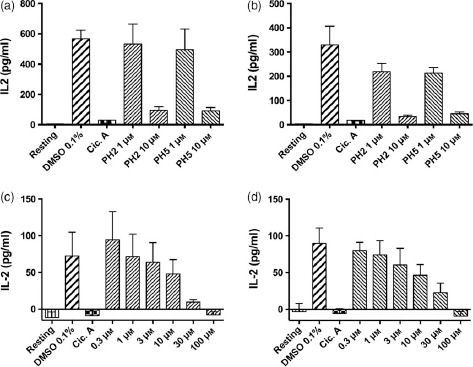
(a) Effect of PH2 and PH5 (1 & 10 μm on inhibition of PMA/PHA-stimulated IL2 release from Jurkat cells. (b) Effect of PH2 and PH5 (1 & 10 μm) on inhibition of anti-CD3/CD28-stimulated IL2 release from Jurkat cells. (c) Effect of PH2 (0.3–100 μm) on inhibition of anti-CD3/CD28-stimulated IL2 release from Jurkat cell. (d) Effect of PH5 (0.3–100 μm) on inhibition of anti-CD3/CD28 stimulated-IL2 release from Jurkat cells. Ciclosporin A at 1 μm. Values expressed as a mean ± SEM (A&B *n* = 3 separate experiments, C&D *n* = 4 separate experiments).

At a concentration of 10 μm, PH2 and PH5 inhibited anti-CD3/CD28-stimulated IL2 release by 86 and 81%, respectively. At 1 μm, inhibition was 8 and 10%, respectively. In comparison, Ciclosporin A at 1 μm inhibited anti-CD3/CD28-stimulated IL2 release by 93%. Both PH2 and PH5 at 10 μm inhibited PMA/PHA-stimulated IL2 release at 10 μm by 80% and by 0.5 and 5%, respectively, at 1 μm. In contrast, Ciclosporin A at 1 μm inhibited PHA/PMA-stimulated IL2 release by 98%, see Figure 2a and 2b. Both compounds PH2 and PH5 (0.3–10 μm) exerted a dose-dependent inhibition of both anti-CD3/CD28-stimulated IL2 release of IL2 from Jurkat cells, Figure 2c and 2d. The latter experiment shows a lower control release of IL-2 and is likely due to experimental variation as a consequence of different passages of Jurkat cells underlying the requirement for internal controls for each experiment. Given that substantial inhibition of IL2 release occurred only at the relatively high concentrations, additional experiments were not carried out and the data were not subjected to statistical analysis as consequence of low values for *n*.

### Dextran sodium sulphate-induced murine colitis

#### Effect of the diastereoisomers, PH2 and PH5

**Figure 3:**
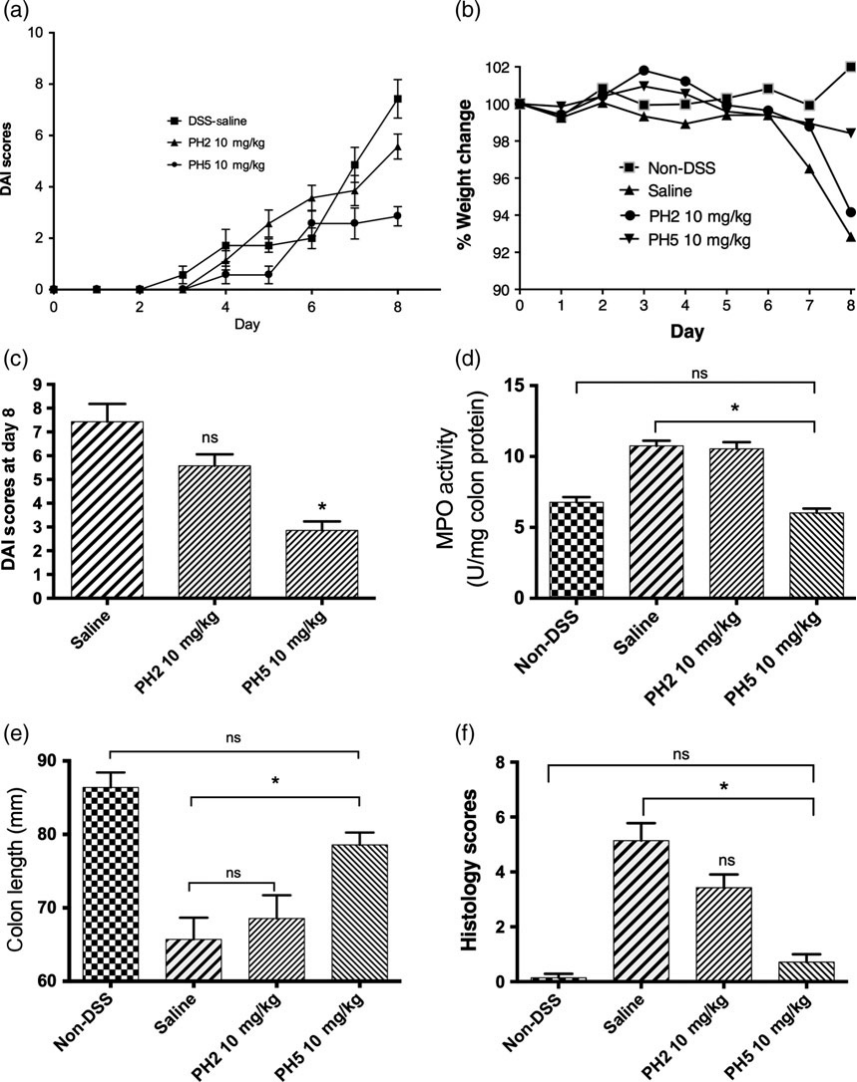
(a) Effect of PH2 and PH5 on disease activity index (DAI) in Murine DSS colitis. (b) Effect of PH2 and PH5 on change in bodyweight. (c) Effect of PH2 and PH5 on DAI at Day 8. (d) Effect of PH2 and PH5 on myeloperoxidase activity at Day 8. (e) Effect of PH2 and PH5 on colon length at Day 8. (f) Effect of PH2 and PH5 on histological damage scores at Day 8. Values expressed as a Mean ± SEM,* n* = 8. *P<0.05. DSS, dextran sulphate sodium.

**Figure 4:**
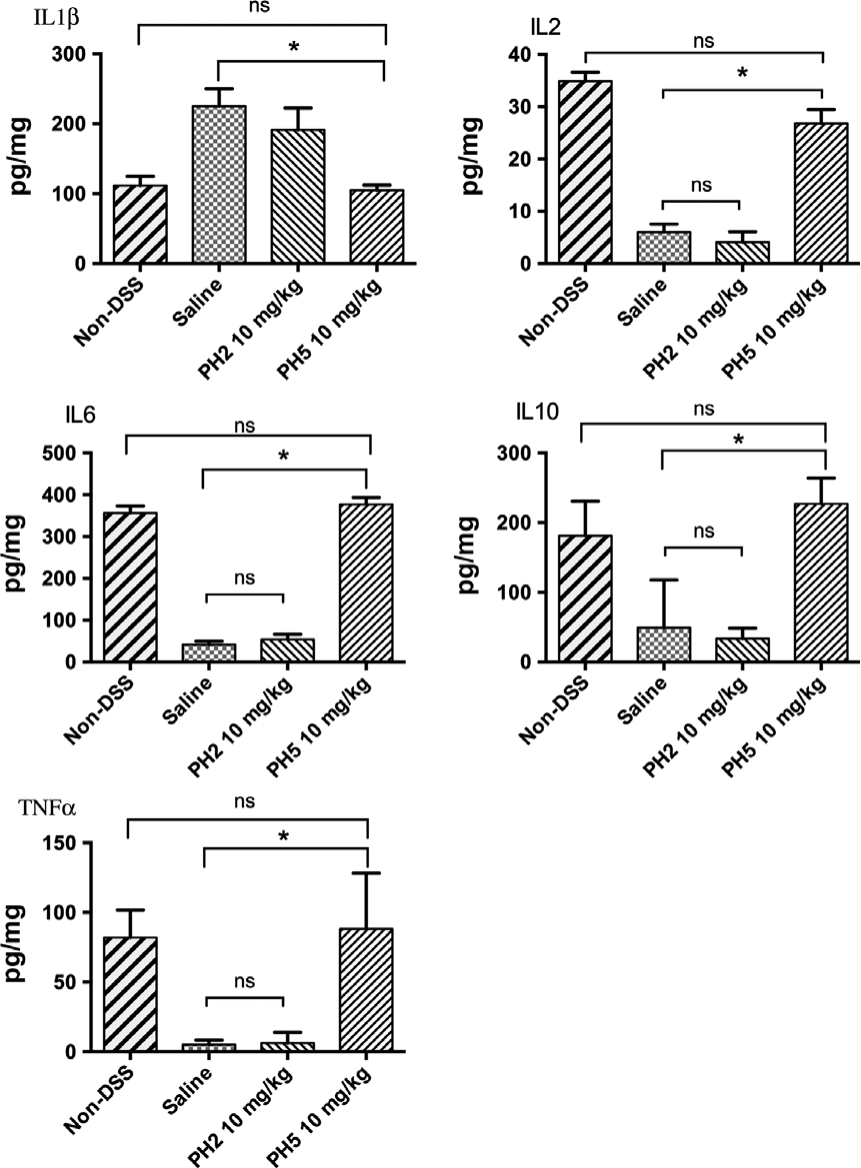
Effect of PH2 and PH5 on colon cytokines at Day 8. Values expressed as a mean ± SEM,* n* = 8. *P<0.05.

Balb/c mice subjected to 5% DSS in the drinking water showed signs of colitis by day 3. This is manifested as an increase in the DAI (Figure 3a) and weight loss (Figure 3b). These symptoms became progressively severe, although in comparison with other experiments in our hands, the colitis was relatively mild and DSS treatment was continued for an additional day. By day 8, the DSS-treated mice had lost up to 10% of their body weight and all mice had profuse rectal bleeding. At autopsy on day 8, there is significant shortening of the length of the colon compared with colons from mice not subjected to DSS (Figure 3e). DAI scores at day 8 were 7.4 ± 0.75. Histology sections of the colon showed the extensive crypt damage and cell infiltration following DSS treatment, the extent of colon damage quantified using an arbitrary scoring system (Figure 3f). Consistent with inflammation of the colon, there was a significant elevation in colon myeloperoxidase (MPO) activity in DSS-treated mice relative to untreated mice (Figure 3d). Quantification of levels of colon cytokines showed that DSS treatment induces elevated IL-1β while other cytokines tested (IL-2, IL-6, IL-10 and TNF-α are reduced (Figure 4).

Mice treated with PH2 and PH5 showed no overt reactions following 7 days of daily intraperitoneal administration of 10 mg/kg. Mice with DSS-induced colitis treated with PH2 (10 mg/kg) developed comparable symptoms of colitis as control saline-treated mice (Figure 3e and 3f). Histological damage scores and MPO levels were not significantly different from vehicle control values. In contrast, mice exposed to DSS and treated with PH5 were protected from colitis. In all parameters tested, PH5-(10 mg/kg) treated mice were significantly less than vehicle control mice but more importantly, were not significantly different from mice that had not colitis induced by DSS (Figure 3a–e). Histology scoring of colon samples confirmed the reduced tissue damage in PH5-treated mice (Figure 3f). The limited colon damage observed on histology sections from PH5 treated mice was also reflected by the reduced levels of colon MPO in PH5 treated mice relative to vehicle control mice (Figure 3d). Furthermore, colon IL-1β, IL-2, IL-6, IL-10 and tumour necrosis factor α (TNF-α) levels in PH5-treated mice were comparable to what was detected in the colons of non-DSS mice; with PH5 and non-DSS mice both having significantly (*P* < 0.05) different colon cytokine levels than vehicle control mice (Figure 4).

#### Effect of the resolved enantiomers of PH5, PH21 and PH22

**Figure 5:**
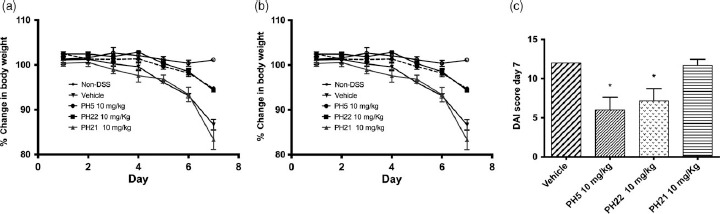
Effect of PH5 and its two resolved enantiomers on (a) change in body weight, (b) Disease activity index (DAI) scores and (c) DAI scores at day 7 in murine DSS colitis. Values expressed as a mean ± SEM,* n* = 7. *P<0.05. DSS, dextran sulphate sodium.

PH5 and its resolved enantiomers, PH21 and PH22 were administered at a dose of 10 mg/kg p.o. to mice with 5% DSS-induced colitis (see Figure 5a–c). In this experiment, the DSS-induced colonic damage was severe, with every mouse of the vehicle control group having a maximum DAI score of 12 at day 7. In contrast, both PH5 and PH22 had the effect of reducing the DAI scores at day 7. PH21 did not significantly reduce DAI scores at day 7. See Figure 5c.

## Discussion

The current report details studies of two novel bi-indane compounds as a pair of diastereoisomers on a variety of *in vivo* and *in vitro* models. We have previously reported that these compounds are equipotent as mast cell stabilisers and have anti-inflammatory properties in the arachidonic mouse ear model.^[12]^ A serendipitous finding was observed in a respiratory model where rats sensitised with intraperitoneal aluminium hydroxide and Freund's adjuvant showed a severe sterile inflammatory peritonitis which was very much reduced by compound PH2 (see Figs. S1–S4). The involvement of Freund's adjuvant suggested we investigate inflammatory responses further, particularly those with a Th_1_-mediated component. Without any knowledge of an affected pathway, our first investigations were *in vivo* using DTH models.

Delayed-type hypersensitivity is a cell-mediated immune response which is protective against intracellular bacteria, fungi and some viruses. However, when inappropriately deployed, it can cause extensive tissue damage in diseases such as rheumatoid arthritis^[20]^ and allograft rejection.^[21]^ Sensitisation to a particular antigen (e.g. *Mycobacterium tuberculosis*) takes place 1 to 2 weeks after first exposure while subsequent exposure to the antigen then stimulates what is thought to be a Th_1_ response.^[22]^ This effector phase occurs within 24 h, peaking after 2 to 3 days. The result is an influx of inflammatory cells, especially macrophages which are the main effector cells in the DTH response.

The mBSA model is a Th_1_ DTH model suitable for examining T cell-mediated immune responses *in vivo*. Sensitisation to a mixture of mBSA and FCA and subsequent challenging with mBSA induces the DTH response. The adjuvant FCA can contain either *M. tuberculosis* or *M*. *butyricum*, both of which are believed to induce the DTH response.^[22–27]^ A second DTH model was the oxazolone-sensitised mouse ear model, a contact hypersensitivity model (CHS), a form of DTH which is very similar to the protein-adjuvant reaction seen in the mBSA model, an inflammatory response to oxazolone challenge in skin, subsequent to prior epidermal sensitisation with the same hapten. Sensitisation to the hapten usually occurs through the skin and involves the binding of hapten to a protein. The resulting complex is antigenic and is processed by APCs. Trafficking of these cells back into the lymph node then occurs, where they will present the antigen to the T cells. Finally, the elicitation phase is largely T cell-mediated, producing IFN-γ and other cytokines, such as IL-17, which further induce expression of additional chemokines and other mediators that lead to increased cellular infiltration.^[28]^

Both PH2 and PH5 were equipotent in the mBSA model and while there is a considerable discrepancy in this model between the doses of the positive control, ciclosporin A, and those of PH2 and PH5, the dose of ciclosporin used falls within literature values^[28,30]^ and increasing the dose of PH2 to 100 mg/kg resulted in a paw swelling of 76 ± 15%, *n*=5, suggesting that a maximal effect occurred at a dose of 10 mg/kg for the compounds. In contrast, PH2 and PH5 were equally ineffective in the oxazolone CHS model. The reasons for this lack of efficacy in this model are unclear; potentially, the fact that compounds were applied topically might have accounted for the difference, although we have previously reported that topical application of these compounds to arachidonic mouse ears was effective in reducing swelling.^[12]^ It therefore seems likely that the inflammatory mechanism in the CHS model differs substantially in respect of the activity our compounds. While the sensitisation stage differs from the mBSA model, one would expect subsequent events to be broadly similar. While IFNγ and TNFα are thought to contribute to CHS by activating macrophages, Th_2_ cytokines suppress Th_1_ cytokine secretion and thus inhibit this response. However, IL-4 may also contribute to CHS as levels of the cytokine were elevated in some oxazolone models.^[14]^

We had sufficient evidence to suggest an interference by PH2 and PH5 in Th_1_ pathways, and an obvious step would be to investigate the effects of the compounds on IL2 release from human T lymphocyte Jurkat cells, a classic calcineurin-mediated Th_1_ *in vitro* model.

Lymphocyte cytokine production is a key to the involvement of these cells in the process of immunity and inflammation. Inhibition of lymphocyte cytokine production and therefore the generation of immune responses is a standard target for many anti-inflammatory agents such as ciclosporin. In these studies, we have examined whether a component of the anti-inflammatory response of PH2 and PH5 seen *in vivo* had any ability to inhibit IL-2 secretion from activated T lymphocytes. IL-2 is a key cytokine that is transcriptionally upregulated by T lymphocytes in response to activation and its synthesis and secretion from these cells contributes to the pathogenesis of many autoimmune and inflammatory diseases. Ciclosporin is the most widely used pharmacological agent to inhibit the synthesis of IL-2. Ciclosporin inhibits IL-2 synthesis by abrogating the activity of an intracellular phosphatase called calcineurin. The inhibition of calcineurin activity prevents the nuclear translocation of the key transcription factor, NF-AT that is involved in upregulation of the IL-2 gene.

Both PH5 and PH2 inhibited IL2 secretion from Jurkat cells stimulated by both TCR stimulation and by PHA/PMA at concentrations which had no significant effect on cell viability. Furthermore, this inhibitory effect was dose dependent in respect of PH2. However, the relative efficacies of the compounds were much less when compared with ciclosporin, by a factor greater than 10-fold. This does not match the relative efficacies observed in the mBSA model, where PH2 and PH5 were of a same similar order of potency as ciclosporin. This suggests that other activities outside of inhibition of Th_1_ cytokines were responsible for a large part of the response in the DTH models.

This notwithstanding, a potent anti-inflammatory effect coupled with a cell-based immunity component suggests a potential use in the treatment of autoimmune inflammatory disease. As a consequence, we investigated the potential efficacy of both PH2 and PH5 in the DSS model of murine colitis.

Dextran sulphate sodium-induced colitis is an experimental mouse model which exhibits many of the symptoms observed in human ulcerative colitis (UC) such as diarrhoea, bloody faeces, mucosal ulceration, shortening of the colon, weight loss and alterations in certain colon cytokines.^[30]^ In these studies, an acute colitis model was used, with 5% DSS administered in the drinking water of Balb/c mice. This dosage regime induces severe acute colitis, by day 7–8 mice will have overt rectal bleeding and marked weight loss; all mice will die by days 10–12 unless sacrificed.

Contrary to expectations, PH5 showed excellent efficacy against all those parameters and variables measured, whereas PH2 had no significant activity against any. In contrast to other similar experiments in our hands, the colitis reported here was relatively mild and mice were sacrificed at day 8 rather than day 7. However, despite this, it is remarkable that mice treated with PH5 at 10 mg/kg appeared not to differ from normal control mice unchallenged by DSS. While colon cytokine levels in PH5-treated mice resembled those of non-DSS mice, the fall in TNFα levels might at first sight appear counter-intuitive. However, it is likely that at day 8, the reduced TNFα levels in vehicle control mice might reflect the morbidity of the colonic tissue.

What was more surprising was the fact that PH2 was ineffective, as in the mBSA model it was equipotent with PH5, and this concurs with our previous work on mast cell mediator release and arachidonic mouse ears, which also showed no difference between the two diastereoisomers.^[12]^ Consequently, this finding suggests that the activity displayed in the DTH model and Jurkat cells are not involved in the protective effect in the DSS murine colitis model. We have also shown in this report that the S,S-enantiomer, PH22 rather than the R,S-enantiomer, PH21 possesses the protective effect against DSS-colitis. Such an enantiomer-specific effect in this model argues for a very specific molecular target, although what system is being targeted by PH5 and PH22 in the DSS-colitis model is presently unknown.

We have previously reported that the glutamine salt of the S,S -enantiomer of the carboxylic acid derivative of PH5, PH46A has good efficacy in both DSS-colitis and the IL10^−/−^ model of spontaneous colitis, models with very different aetiologies.^[32]^ Furthermore, PH46A does not inhibit IL2 release from Jurkat cells (see Fig. S5). This gives further credence to the concept that the beneficial effect of these compounds in colitis models is unrelated to any effect on T cells or Th_1_ cytokines, but is potentially a new and previously unexploited target in this disease model. Investigations into what this target might be are ongoing and may point to a novel therapeutic approach to the treatment of inflammatory bowel disease.

## Author Contributions

N H Frankish: main author, co-director of research program. Brendan McHale: contributed to laboratory work. Helen Sheridan: co-author, co-director of research program.

Declarations

## Supplementary Material

jphp12846-sup-0001-FigS1-S5 **Figure S1**. Livers from sensitised (Freunds, Aluminium Hydroxide and ovalbumin, I.p.) but unchallenged rats, showing sterile peritonitis as a consequence of the immunological insult.
**Figure S2**. Livers from sensitised (Freunds, Aluminium Hydroxide and ovalbumin, I.p.) rats, challenged with ovalbumin aerosol showing sterile peritonitis as a consequence of the immunological insult.
**Figure S3**. Livers from sensitised (Freunds, Aluminium Hydroxide and ovalbumin, I.p.) rats, challenged with ovalbumin aerosol and treated with cromoglycate by aerosol, showing sterile peritonitis as a consequence of the immunological insult.
**Figure S4**. Livers from sensitised (Freunds, Aluminium Hydroxide and ovalbumin, I.p.) rats, challenged with ovalbumin aerosol and treated with PH2 by aerosol (six doses), with sterile peritonitis (as a consequence of the immunological insult) being completely absent.
**Figure S5**. Effect of PH46 (30 nm–100 µm) and ciclosporin A at 1 μm on inhibition of anti-CD3/CD28 stimulated IL2 release from Jurkat cells. Values expressed as a mean ± SEM, *n* = 3 separate experiments.
