# Supplementary material for: The indane diastereoisomers, PH2 and PH5: divergence between their effects in delayed-type hypersensitivity models and a model of colitis
Source: J Pharm Pharmacol. 2017 Oct 23;70(1):101–10. doi: 10.1111/jphp.12846 (PMC5887892; doi:10.1111/jphp.12846)
Supplement: jphp12846-sup-0001-FigS1-S5 — Figure S1. Livers from sensitised (Freunds, Aluminium Hydroxide and ovalbumin, I.p.) but unchallenged rats, showing sterile peritonitis as a consequence of the immunological insult. Figure S2. Livers from sensitised (Freunds, Aluminium Hydroxide and ovalbumin, I.p.) rats, challenged with ovalbumin aerosol showing sterile peritonitis as a consequence of the immunological insult. Figure S3. Livers from sensitised (Freunds, Aluminium Hydroxide and ovalbumin, I.p.) rats, challenged with ovalbumin aerosol and treated with cromoglycate by aerosol, showing sterile peritonitis as a consequence of the immunological insult. Figure S4. Livers from sensitised (Freunds, Aluminium Hydroxide and ovalbumin, I.p.) rats, challenged with ovalbumin aerosol and treated with PH2 by aerosol (six doses), with sterile peritonitis (as a consequence of the immunological insult) being completely absent. Figure S5. Effect of PH46 (30 nm–100 µm) and ciclosporin A at 1 μm on inhibition of anti-CD3/CD28 stimulated IL2 release from Jurkat cells. Values expressed as a mean ± SEM, n = 3 separate experiments. [file jphp12846-sup-0001-FigS1-S5.docx]

# Supplementary material

**Fig. S1**

**Fig. S2**

**Fig S3**.

**Fig. S4**

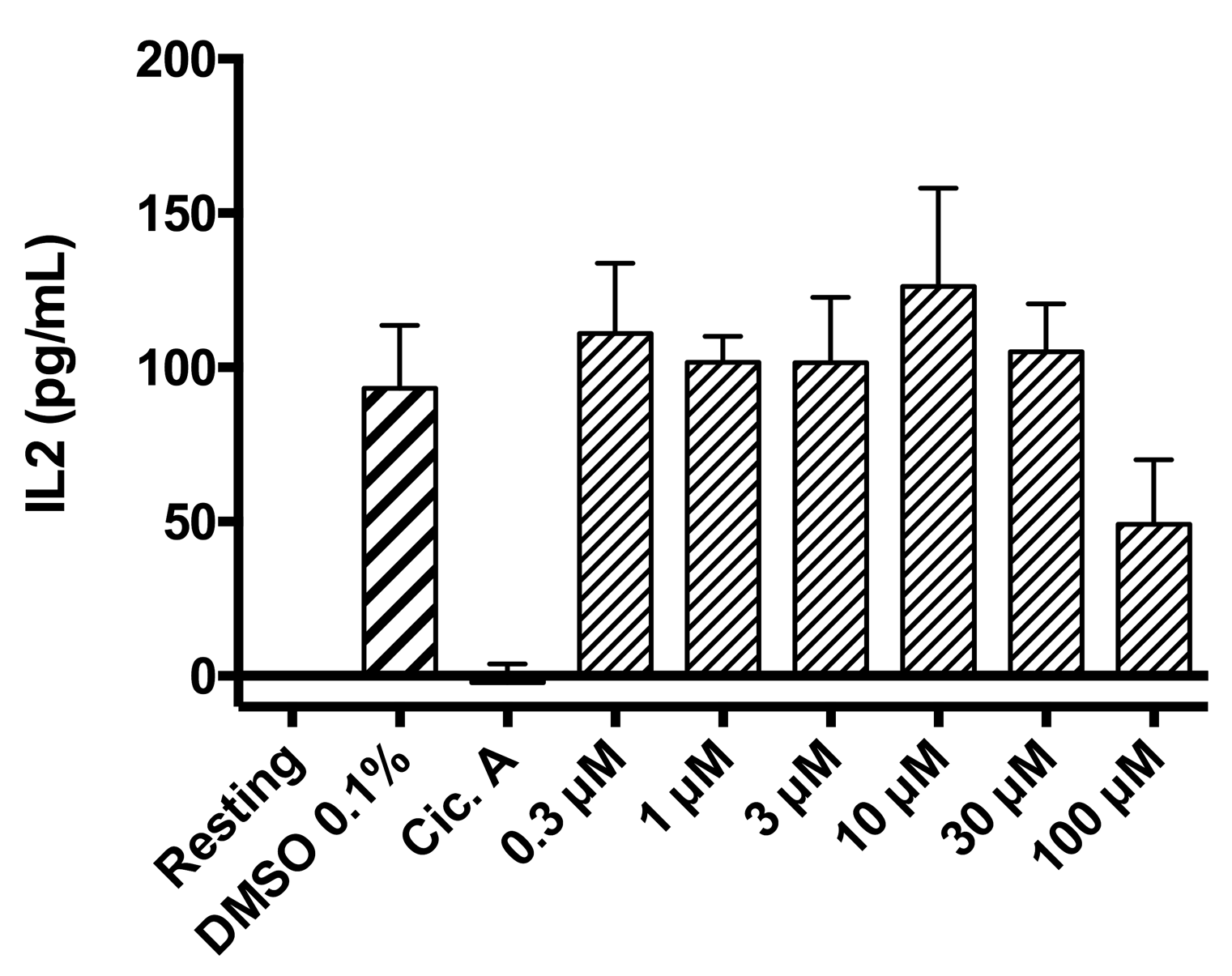


**Fig S5** Effect of PH46 (30nM - 100 µM) and ciclosporin A at 1 μM on inhibition of anti-CD3/CD28 stimulated IL2 release from Jurkat cells. Values expressed as a mean ± SEM, n= 3 separate experiments
